# A drug interaction study investigating the effect of Rifabutin on the pharmacokinetics of Maraviroc in healthy subjects

**DOI:** 10.1371/journal.pone.0223969

**Published:** 2019-10-24

**Authors:** M. Ghannad, M. Dennehy, C. la Porte, I. Seguin, D. Tardiff, R. Mallick, E. Sabri, G. Zhang, S. Kanji, D. W. Cameron

**Affiliations:** 1 Ottawa Hospital Research Institute, Ottawa, Ontario, Canada; 2 Clinical Investigation Unit, The Ottawa Hospital, Ottawa, Ontario, Canada; 3 Ottawa Methods Centre, Clinical Epidemiology Program, Ottawa Hospital Research Institute, Ottawa, Ontario, Canada; 4 The Ottawa Hospital, Department of Pharmacy, Ottawa, Ontario, Canada; 5 Clinical Epidemiology Program, Ottawa Hospital Research Institute, Ottawa, Ontario, Canada; 6 Division of Infectious Diseases, Department of Medicine, University of Ottawa at The Ottawa Hospital, Ottawa, Ontario, Canada; Azienda Ospedaliera Universitaria di Perugia, ITALY

## Abstract

Effects of steady-state rifabutin on the pharmacokinetics of steady-state maraviroc were investigated in fourteen healthy adult female and male volunteers. Maraviroc 300 mg twice daily (BID) was given orally with food for fifteen days. On day six, rifabutin 300 mg once daily (QD, P.O.) was added to the regimen. Formal pharmacokinetic (PK) sampling was performed on days five and fifteen. Individual plasma drug concentration-time data for maraviroc, and rifabutin on day fifteen, were obtained using validated High Performance Liquid Chromatography (HPLC) tandem Mass Spectrometry (MS/MS). Rifabutin steady state exposure was comparable to data in the literature. Maraviroc area under the curve (AUC) and minimum plasma concentration (C_last_ or C_min_) were reduced by 17% and 30% respectively when co-administered with rifabutin. No unexpected or serious adverse eventsoccurred. Based on the reduced exposure of maraviroc observed in this study, increasing the dose of maraviroc may be studied to normalize its moderately reduced exposure following rifabutin co-administration, a moderate inducer of CYP3A4.

## Introduction

Immunocompromised HIV patients are susceptible to invasive comorbid conditions and often require combination drug therapy. These patients can be limited in the choice of appropriate therapies and are prime candidates for complex drug/drug interactions. Maraviroc, often coadministred with other drugs in patients with HIV, is a CCR5 chemokine co-receptor antagonist that selectively and reversibly prevents the interaction of HIV-1 gp120 with the CCR5 receptor, inhibiting the conformational changes required for CCR5 tropic HIV-1 to enter CD4 cells and multiply.[1] Maraviroc is primarily metabolized by cytochrome P450 3A4 (CYP3A4) enzyme, and is also a substrate for transporters such as p-glycoprotein, OATP1B1 and SLCO1B1.[1] This small antiretroviral molecule has an absolute bioavailability of approximately 33% following the approved standard twice a day oral dose of 300 mg and has a half-life of 14–18 hours in healthy subjects.[2–4] The effect of food was investigated in a formal pharmacokinetic study, and it was observed that food decreased the AUC, but not the C_min_.[3–5] Similarly, no significant difference was observed in the viral load reduction in the fed and unfed state, and therefore maraviroc may be administered with or without food.[5] Maraviroc has no net inhibitory or inductive effect on CYP enzymes. Drugs that inhibit or induce CYP3A4, however, have the potential to significantly alter the maraviroc pharmacokinetic profile. As a result, the maraviroc dose may need to be adjusted when co-administered with potent inhibitors or inducers of CYP3A4.[1,2,5] This highlights the need to investigate the potential interactions of drugs that influence CYP3A4 activity, especially those commonly co-administered in the treatment of HIV-infected patients.

Rifampin and rifabutin, both rifamycin derivatives, have demonstrated clinical efficacy against *Mycobacterium tuberculosis* and *Mycobacterium avium‐intracellulare* complex (MAC) infections often treated in HIV patients.[6] A drug-drug interaction study of maraviroc found that when co-administered with rifampin, a potent CYP3A inducer, a 63% decrease in exposure was observed, requiring doubling of the maraviroc dose to compensate for this effect.[7] Given the potent CYP induction by rifampin, rifabutin is sometimes prescribed as an alternative for the prevention and treatment of tuberculosis and non-tuberculosis mycobacterial infections.[8] Rifabutin is a moderate inducer and also a substrate for CYP3A4.[9,10] Like the parent compound, the 25-O-desacetyl-rifabutin metabolite has activity and contributes up to 10% of the total anti-bacterial activity. Although rifabutin is a less potent inducer of CYP3A than rifampin, maraviroc exposure is expected to decrease as a result of the interaction with rifabutin, and an increase in the maraviroc dose when co-administered with rifabutin may be necessary. Maraviroc was not expected to inhibit the metabolism of rifabutin as maraviroc does not have a net inhibitory effect on CYP3A4.[3]

The objective of the current study was to evaluate the potential for a drug interaction when co-administering maraviroc 300 mg twice daily (BID) and rifabutin 300 mg once daily (QD) in healthy adults, and the secondary outcomes were to determine the safety and tolerability of these agents in combination. The primary pharmacokinetic (PK) outcomes were area under the concentration-time curve (AUC_12_), maximum concentration of drug in plasma (C_max_), and concentration at 12 h post-dose (C_12_ or C_last_) for maraviroc without and with rifabutin, as well as the AUC, C_max_, and the concentration at 24 h post-dose (C_24 or_ C_last_) for rifabutin and its major metabolite, 25-O-desacetyl-rifabutin. The secondary outcomes were adverse events and routine clinical safety lab results.

## Materials and methods

### Study design

This study protocol was approved by the Ottawa Hospital Research Ethics Board (OHREB) and performed at the Ottawa Hospital Research Institute (OHRI Protocol #: 20130080-01H). The trial was performed in compliance with the Canadian Tri‐Council Policy Statement version 2 (TCPS2), the World Medical Association Declaration of Helsinki (October 2000), the Canadian Food and Drug Regulations, Division 5 Part C, the Canadian Ontario's Personal Health Information. Protection Act (PHIPA), and the ICH Good Clinical Practices (GCP). Study results are reported in accordance with the ClinPK reporting guidelines.[11] All participants gave their informed written consent prior to enrolment. The study was carried out unblinded.

Eligible subjects were healthy male and female participants between 18 and 65 years of age, with a body mass index (BMI) between 17.5 to 30.5 kg/m^2^, and a total body weight of >50 kg (110 lbs). Other inclusion criteria included having acceptable medical history, physical examination, and 12‐lead ECG at screening, as well as adequate baseline organ function indicated by laboratory values. Participants were required to abstain from alcohol for three days prior to and during the study. Participants were excluded if they had serological evidence of exposure to HIV or used any prescription or non-prescription medications (with the exception of occasional use of acetaminophen up to 1000 mg per day) within 2 weeks prior to or during the study period. Female subjects were excluded if they were pregnant, breastfeeding or not using a barrier method of birth control.

**Fig 1:**
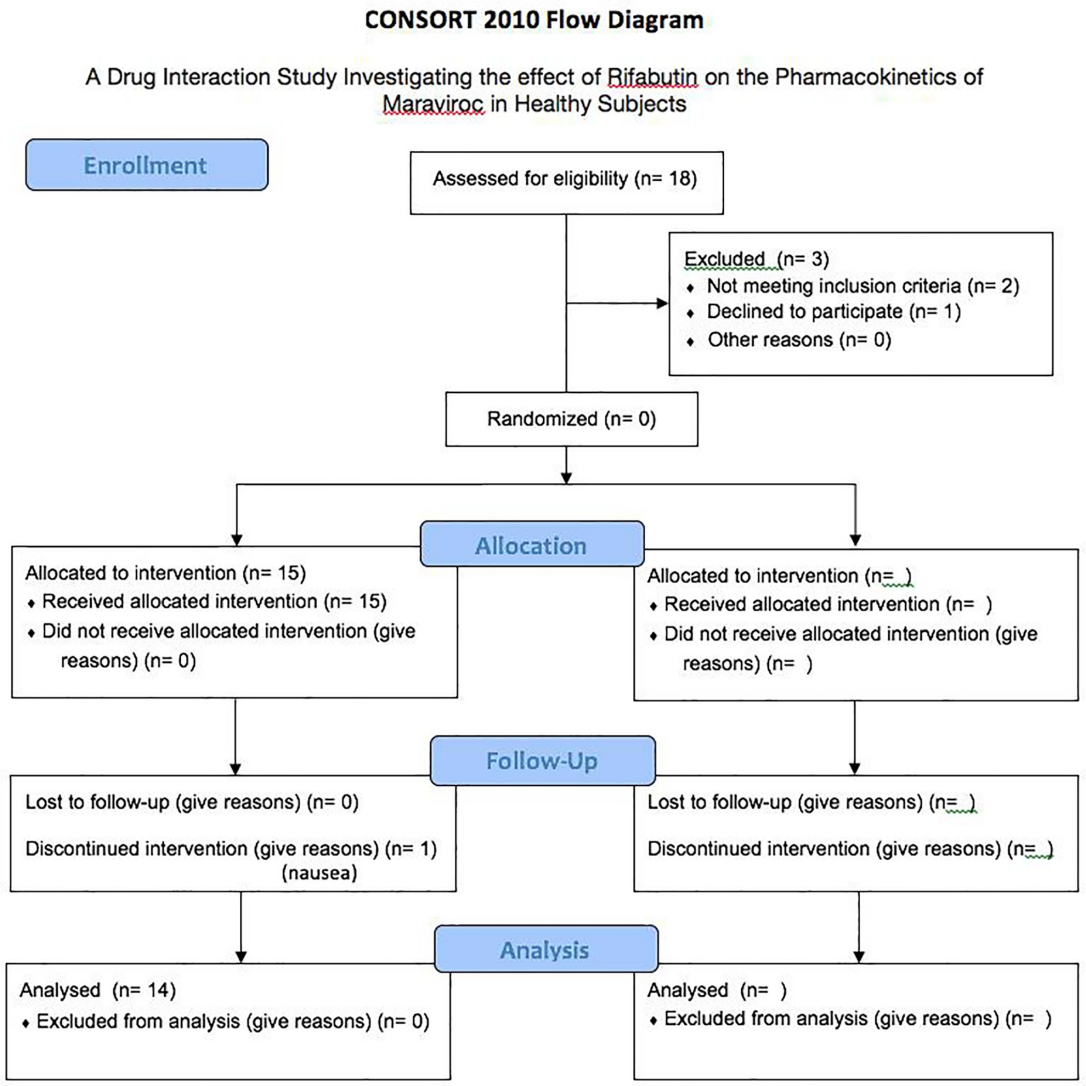
CONSORT flow diagram [12].

**Fig 2:**
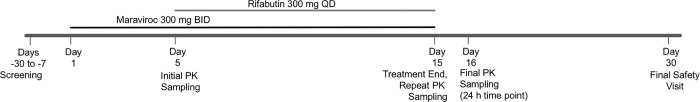
Study timeline.

The Clinical Investigation Unit (CIU) at the OHRI has a database of healthy volunteers interested in participating in clinical studies who were contacted and recruited by phone or email. The first patient was recruited on 06 Sept 2013 and the last follow-up visit was on 23 Dec 2013. A description of participant recruitment can be found in the CONSORT flow diagram in Fig 1 and a summary of the study timeline can be found in Fig 2.

Participants received 300 mg of maraviroc BID orally at 8:00 a.m. (with breakfast) and 8:00 p.m. (with a snack) on days 1 to 15 in groups of 1–4 individuals. On days 6 to 15, rifabutin at 300 mg QD was added to the treatment regimen as a single dose to be taken orally at the same time in the morning as maraviroc. The treatment period ended on day 15, with the last PK sample drawn on day 16. Clinical staff observed the administration of the morning dose on Days 1, 3, 8, 10, and 12 as well as the morning and evening doses on Days 5 and 15. On days that participants were self-dosing at home they were instructed to record times of drug intake in a diary. Meals were standardized on PK study days (days 5 and 15).

On study day 5, 12 hour PK sampling of maraviroc was performed just before the morning dose of maraviroc and at 0.5, 1, 1.5, 2, 3, 4, 5, 6, 8, 10 and 12 hours after dosing with a standardized meal. On day 15, 12 hour PK sampling was repeated. Subjects returned on day 16 to draw a 24 hour post-dose sample for rifabutin pharmacokinetic measurements. The trial ended on day 30 after a final safety follow-up visit.

### Bioanalysis

Plasma samples were analyzed for maraviroc, rifabutin, and 25‐O‐desacetyl-rifabutin concentrations using a validated High Performance Liquid Chromatography (HPLC) tandem Mass Spectrometry (MS/MS) method.[13,14] Samples were pre‐treated with methanol to obtain protein precipitate, after which the supernatant was used for injection. A multipoint (n ≥ 6) calibration curve was generated for each analytical run, which was subsequently used to calculate the concentration of the compounds of interest in the samples. A set of Quality Control samples (low, medium, and high) were analyzed at the beginning and end of each analytical run.

### Pharmacokinetics

Noncompartmental methods were used for PK analysis. The highest observed plasma concentration was defined as C_max_, with the corresponding sampling time as t_max_. The elimination rate constant (λz) was estimated using linear regression analysis (log C versus t) and the selection was based on best-adjusted square coefficient of regression (r^2^). A minimum of three points were needed for this estimate. The AUC was estimated using the linear-log trapezoidal rule (linear up/log down) for 0 to 12 h post-dose for maraviroc and 0 to 24 h post-dose for rifibutin and its metabolite. The maraviroc concentration at 12 h post-dose was defined as C_12_ or C_last_ and that of rifabutin and its metabolite, 25-O-desacetyl-rifabutin, defined as C_24_ or C_last_. The average maraviroc concentration (C_avg_) was defined as AUC_0-12_/12. Apparent total-body clearance (Cl/F) was calculated as the ratio of dose to AUC, where F represents the oral bioavailability, and the volume of distribution (V_d_) was calculated as (Cl/F)/λz.

### Safety

Safety evaluations including AEs reported to study staff and/or entered in their diaries, laboratory biochemistry data, and physical exams were documented throughout the study.

### Statistical analysis

Computation of power estimates based on previous antiretroviral pharmacokinetic studies, suggest that a sample size of 12 (assuming a 40% coefficient of variation) is adequate for the detection of a 35% difference in the AUC for maraviroc.[15] Geometric means and coefficients of variation were calculated for all PK parameters for both reference treatment (maraviroc alone) and test treatment (maraviroc plus rifabutin). The ratio of the geometric means of the maraviroc AUC, C_max_, and C_12_ for maraviroc plus rifabutin to those for maraviroc alone along with their 90% confidence intervals were calculated. All pharmacokinetic and statistical analysis was performed with SAS (SAS Institute Inc., Cary, NC: release 9.1).

## Results

Of the eighteen subjects recruited, 3 were excluded for either not meeting the inclusion criteria (2) or declining to participate (1). Fifteen subjects were allocated to the treatment with one patient discontinuing due to nausea. The data from fourteen subjects were analyzed.

### Pharmacokinetics

Fourteen patients were included in the pharmacokinetic analysis, 50% of whom were male. Participants had a mean age of 33 years and a mean body mass (total body weight) of 75 ± 13 kg. The mean maraviroc systemic exposure was reduced with concomitant administration of rifabutin. Systemic exposure parameters and geometric mean plasma pharmacokinetic data of maraviroc and in combination with rifabutin are summarized in Table 1.

**Table 1:** Summary of steady-state pharmacokinetics for Maraviroc^a^.

PK Parameter	Maraviroc alone	Maraviroc + Rifabutin	GMR (90% Cl)
AUC (h*μg/L)	1026.2 (73.6)	847.0 (66.6)	0.83 (0.70–0.97)
C_max_ (μg/L)	304.6 (91.1)	239.8 (96.6)	0.79 (0.57–1.09)
C_last_ or C_12_ (μg/L)	23.3 (45.5)	16.3 (45.5)	0.70 (0.59–0.82)
T_max_	1.2 (1.0–4.0)	2.0 (1.0–5.0)	
Cl/F (L/h)	235.0 (62.2)	319.8 (62.2)	
V_d_/F (L)	1833.6 (132.7)	1496.3 (85.2)	

^a^ Values shown are geometric means, with the geometric coefficient of variation (%), except for the T_max_ value which is a median (min-max). AUC, area under the curve; C_max_, maximum plasma concentration; C_last_ or C_12_, minimum concentration at last (12 hour) data point; T_max_, time at which maximum plasma concentration was reached; Cl, clearance; F, bioavailability; V_d_, volume of distribution.

**Fig 3:**
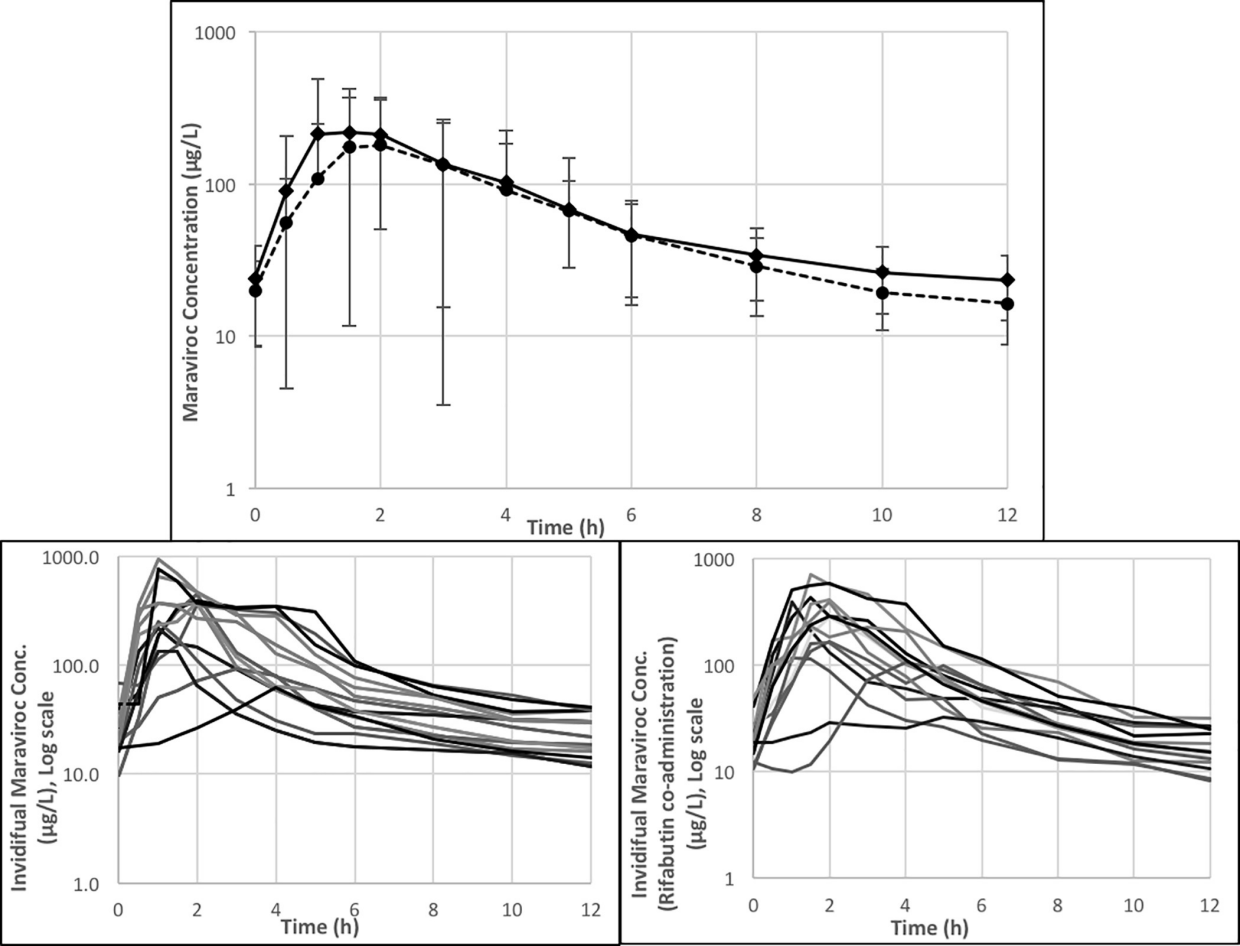
Steady-state geometric mean Maraviroc concentration-time profile (log-scale) for subjects receiving Maraviroc alone (squares and solid line) and Maraviroc plus Rifabutin (circles and dashed line)(A). Steady-state maraviroc concentration-time profile (log-scale) for individual participants without (B) and with rifabutin (C) co-administration.

With rifabutin co-administration, the geometric mean C_last_ for maraviroc went from 23.3 ug/L to 16.3 μg/L, a 30% decrease compared to that of maraviroc alone (Fig 3, Table 1). The mean maraviroc AUC, C_max_ and C_avg_ decreased in the presence of rifabutin. The AUC decreased by 17% going from 1026 μg*h/L to 847 μg*h/L, C_max_ by 21% going from 305 μg/L to 240 μg/L, and C_avg_ by 18% going from 85 μg/L to 70 μg/L. The geometric mean ratio (GMR) and 90% confidence interval for AUC, C_max_, and C_last_ comparing maraviroc alone and in combination with rifabutin were 0.83 (0.70–0.97), 0.79 (0.57–1.09), and 0.70 (0.59–0.82), respectively. In the presence of rifabutin, the maraviroc concentrations were lower in the absorption and elimination phases of the steady-state time-concentration profile as compared to when maraviroc was administered alone (Fig 3A). Plots of the invidual maraviroc concentration-time profiles without and with rifabutin co-administration can be seen in Fig 3B and 3C respectively.

**Fig 4:**
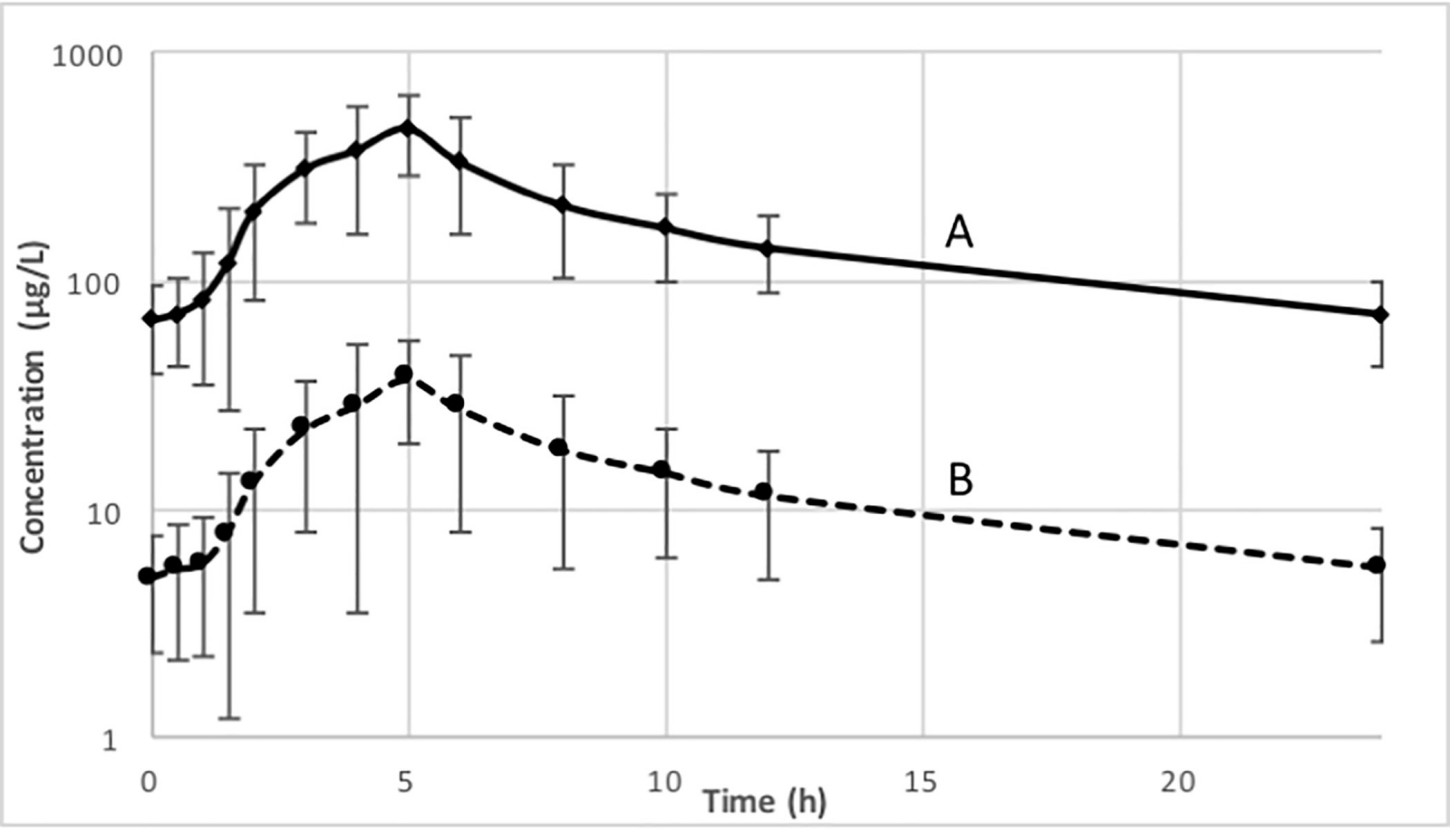
Steady-state geometric mean (log-scale) Rifabutin (A) and 25-O-desacetyl Rifabutin (B) concentration-time profile for participants receiving Maraviroc and Rifabutin.

**Fig 5:**
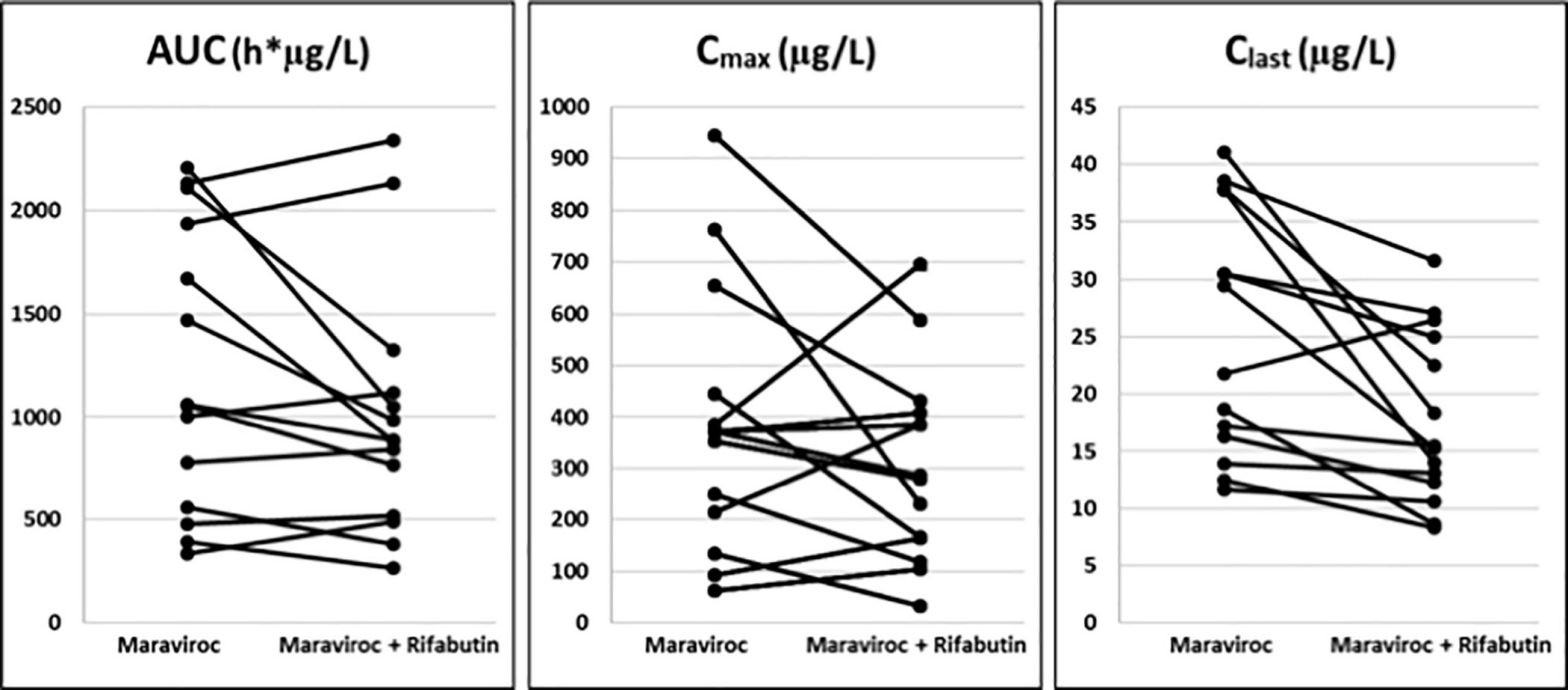
Effect of steady-state Rifabutin on the steady-state Maraviroc AUC, C_max_, C_last_ for individual subjects.

The geometric mean drug concentration-time profile for rifabutin and 25-O-desacetyl-rifabutin can be found in Fig 4, with the rifabutin metabolite remaining present at 10% that of the parent drug. Pharmacokinetic parameters of rifabutin and 25-O-desacetyl-rifabutin are summarized in Table 2 and again, the metabolite is present at roughly 10% that of rifabutin with comparable pharmacokinetic parameters. The individual effect of rifabutin on the maraviroc AUC, C_max_, and C_last_ be seen in Fig 5. The overall trend is a decrease in parameters, although 6 out of 14 participants, actually had a slight increase in both AUC and C_max_ and one participant in C_last_.One participant had very low levels of maraviroc throughout the measured period and was not excluded from our 14-patient sample.

**Table 2:** Summary of the steady-state pharmacokinetics for Rifabutin and 25-O-desacetyl rifabutin (Day 15)^a^.

PK parameter	Maraviroc + Rifabutin
Rifabutin	
AUC (h*μg/L)	4,221.9 (35.6)
C_max_ (μg/L)	542.2 (33.5)
C_last_ or C_24_ (μg/L)	71.2 (42.8)
T_max_	5.0 (3.0–5.0)
Cl/F (L/h)	54.7 (39.2)
V_d_/F (L)	925.1 (34.6)
25-O-desacetyl rifabutin	
AUC (h*μg/L)	331.9 (53.6)
C_max_ (μg/L)	42.3 (53.9)
C_last_ or C_24_ (μg/L)	5.5 (51.3)
T_max_ (h)	5.0 (3.0–6.0)
Cl/F (L/h)	714.2 (52.3)
V_d_/F (L)	10,817 (57.8)

^a^ Values shown are geometric means, with the geometric coefficient of variation (%), except for T_max_ which is a median (min-max). AUC, area under the curve; C_max_, maximum plasma concentration; C_last_ or C_12_, minimum concentration at last (24 hour) data point; T_max_, time at which maximum plasma concentration was reached; Cl, clearance; F, bioavailability; V_d_, volume of distribution.

### Safety

One subject discontinued treatment before PK analysis due to AEs (nausea and headache) and was replaced. All 14 subjects who received study medications were included in the safety evaluation. No deaths or serious AEs occurred and no significant changes in vital signs or physical findings were observed during this study. When taking both maraviroc and rifabutin, 5/14 (35.7%) participants reported headache, 2/14 (14.2%) fever, 2/14 (14.2%) joint pain, and 1/14 (7.1%) muscle pain. The only adverse event reported with maraviroc alone was headache, 2/14 (14.2%). Table 3 summarizes the safety data for this study.

**Table 3:** AEs and laboratory abnormalities.

	Maraviroc	Maraviroc + Rifabutin	Total
No. of subjects (%)	14 (100.0)	14 (100.0)	14 (100.0)
Total No. with adverse events, N (%)	6/14 (50.0)	7/14 (50.0)	11/14 (78.6)
No. with mild AE, N (%)	0	5/14 (35.7)	3/14 (21.4)
Joint pain	0	2	2/14 (14.3)
Fever	0	2	2/14 (14.3)
No. with moderate AE, N (%)	2/14 (14.3)	7/14 (50.0)	8/14 (57.1)
Headache	2	4	6/14 (42.8)
Muscle pain	0	1	1/14 (7.1)
No. with severe AE, N (%)	0	0	0
No. AST abnormality (%)	1/14 (7.1)	1/14 (7.1)	2/14 (14.3)
No. with AST grade 3 or 4 (%)	0	0	0
No. ALT abnormality (%)	0	2/14 (14.3)	2/14 (14.3)
No. with ALT grade 3 or 4 (%)	0	0	0
No. WBC abnormality (%)	0	9/14 (64.3)	9/14 (64.3)
No. with WBC grade 1 or 2 (%)	0	2/14 (14.3)	2/14 (14.3)
No. with WBC grade 3 or 4 (%)	0	0	0
No. Platelets abnormality (%)	1/14 (7.1)	3/14 (21.4)	3/14 (21.4)
No. with platelets grade 1 or 2 (%)	0	1/14 (7.1)	1/14 (7.1)
No. with platelets grade 3 or 4 (%)	0	0	0
No. Lymphocytes abnormality (%)	0	5/14 (35.7)	5/14 (35.7)
No. Lymphocytes grade 1 or 2 (%)	0	0	0
No. Lymphocytes grade 3 or 4 (%)	0	4/14 (28.6)	4/14 (28.6)
No. Neutrophils abnormality (%)	0	6/14 (42.8)	6/14 (42.8)
No. Neutrophils grade 3 or 4 (%)	0	1/14 (7.1)	1/14 (7.1)

## Discussion

Rifampin, a rifamycin, has been shown to be a net inducer of CYP3A4 and decreases the overall systematic exposure of maraviroc when co-administered.[7] Another rifamycin, rifabutin, was hypothesized to also lead to a decrease in systemic exposure of maraviroc in a combination treatment since maraviroc is a CYP3A4 substrate and rifabutin is both a moderate inducer and a substrate for CYP3A4.[16,17]

The co-administered of maraviroc and rifabutin in healthy adults did lead to a decrease in the overall exposure of maraviroc with AUC decreasing by 17%, C_last_ by 30% and C_avg_ by 18% in steady-state. In some patients, this moderate reduction in maraviroc concentration may necessitate an increase in dose of maraviroc to maintain the same exposure when co-administered with rifabutin, as recommended for rifampin.

Maraviroc concentrations measured in this study varied greatly from patient to patient and were lower on average than those published.[1] High interpatient variability in systemic maraviroc concentration was also observed in a study performed by Lu *et al* with the same dose of maraviroc.[18] This variability may be due to inherent individual genetic differences in endogenous metabolizing enzymes and cellular transporters for which maraviroc is a substrate. Conversely, the rifabutin concentrations were more consistent between participants and similar to those published in the literature indicating rifabutin may be less susceptible to putative differences in metabolizing enzymes and cellular transporters.[13] The main focus of this work, however, was on changes in maraviroc concentrations due to the introduction of the second drug, rifabutin, rather than on the absolute levels themselves. Future studies could include a larger number of participants to clarify the observed maraviroc variability and its impact on clinical outcomes such as viral load.

The antiretroviral activity of drugs such as protease inhibitors and non-nucleoside reverse transcriptase inhibitors are known to have a good correlation with plasma and intracellular concentration. Maintaining plasma drug concentrations above a given trough level (C_min_ or C_last_) of 50 ug/L is an NIH accepted indicator in the field of drug efficacy due to equilibration of intra- and extra-cellular drug levels.[1] Our C_min_ values (23 ug/L and 16 ug/L for maraviroc and maraviroc plus rifabutin respectively) were below this cut-off which may have been due to the inherent variability in participant maraviroc drug metabolism. C_avg_ has also been used as an indicator of maraviroc efficacy with a cut-off of 100 ug/L[19,20], derived from a sub-study of the MOTIVATE trial, and of 75 ug/L[14] for treatment-naïve patients. The C_avg_ values for maraviroc found in this study fall within that range at 85 ug/L and for maraviroc plus rifabutin, just below at 70 ug/L. These lower values may also may be due to the genetic variability discussed above. Additionally, C_avg_ is a measure of AUC (AUC_0-12_/12) which is known to decrease when maraviroc is taken with food which it was in this study.

A similar effect of maraviroc on rifabutin was not expected as maraviroc is not a known inhibitor or inducer of CYP3A4 and therefore unlikely to modify the metabolism of rifabutin or its metabolite, 25-O-desacetyl-rifabutin.[3] Given that rifabutin and its active metabolite have similar antibacterial activity, the overall AUC of rifabutin and 25-O-desacetyl-rifabutin is a preferred indicator of drug activity.[21] This combined rifabutin AUC value in our study was 4554 h*μg/L which is comparable to other combined daily steady-state AUC values adjusted to the 12 hour dosing time frame for 300 mg of rifabutin administered alone in the literature (AUC at 12 hours: 2090–3380 h*μg/L).[13]

Both maraviroc and rifabutin are well tolerated individually,[22] and no serious AEs were observed with their combination within this study. Headache, muscle pain, joint pain, fever, and mild reversible liver enzyme abnormalities were the most commonly reported adverse drug reactions. Strengths of this study include observed dosing and standardized meals which assured timing adherence on PK days.

Our findings indicate that rifabutin has the effect of decreasing the mean systemic exposure of maraviroc in healthy HIV-negative adults. Increasing the maraviroc dose may normalize the average exposure to that observed without rifabutin co-administration, as with rifampin. Care should be taken when prescribing maraviroc, however, as in some cases, standard dosing may be sufficient in stable patients. It is important to note that since all subjects were healthy volunteers, any possible dose adjustment required may be different in HIV patients who would most likely receive co-administration of drugs such as maraviroc with other anti-retrovirals and/or protease inhibitors that interact with CYP3A4. Furthermore, HIV patients’ rate of drug absorption may be affected by their HIV status also altering the effective dose required.[23] Future studies should investigate systemic exposure in persons with HIV at standard and higher doses of maraviroc co-administered with rifabutin.

## Other information

The trial is registered at ClinicalTrials.gov.no. NCT01894776, https://clinicaltrials.gov/ct2/show/NCT01894776?term=A+Drug+Interaction+Study+Investigating+the+effect+of+Rifabutin+on+the+Pharmacokinetics+of+Maraviroc+in+Healthy+Subjects&rank=1

Maraviroc capsules and Rifabutin tablets were provided by ViiV Healthcare, a Pfizer Inc. affiliate.

## Supporting information

S1 Table ClinPK checklist for pharmacokinetic study reporting.[11]. (DOCX)

S2 Table Raw LCMSMS data for maraviroc (with and without rifabutin co-administration), rifabutin, and 25-O-desacetyl-rifabutin. (XLSX)

S3 Table TREND checklist. (PDF)

S1 Text Study protocol. (PDF)
