# Supplementary material for: A drug interaction study investigating the effect of Rifabutin on the pharmacokinetics of Maraviroc in healthy subjects
Source: PLoS One. 2019 Oct 24;14(10):e0223969. doi: 10.1371/journal.pone.0223969 (PMC6812819; doi:10.1371/journal.pone.0223969)
Supplement: S1 Table — (DOCX) [file pone.0223969.s001.docx]

**Table 4: ClinPK checklist for pharmacokinetic study reporting.**
