# Supplementary material for: A drug interaction study investigating the effect of Rifabutin on the pharmacokinetics of Maraviroc in healthy subjects
Source: PLoS One. 2019 Oct 24;14(10):e0223969. doi: 10.1371/journal.pone.0223969 (PMC6812819; doi:10.1371/journal.pone.0223969)
Supplement: S1 Text — (PDF) [file pone.0223969.s004.pdf]

STUDY TITLE:

A drug interaction study investigating the effect of rifabutin on the pharmacokinetics of maraviroc

Protocol Version 4.2
Date of version: May 09, 2013

OHRI Protocol #: 2013 0080-01H

INVESTIGATOR(S):
D.William Cameron, MD, FRCPC, Ottawa Hospital Research Institute
Charles la Porte, PharmD, PhD,

ADDRESS: 501 Smyth Road, mailbox 228
TELEPHONE: 613-737-8899 ext 78923
FAX: 613-737-8925
E-MAIL ADDRESS: bcameron@ohri.ca

PROTOCOL SIGNATURE PAGE

A drug interaction study investigating the effect of rifabutin on the pharmacokinetics of maraviroc

Version: 4.2

My signature below confirms that I have reviewed and approved this protocol prior to submission to Health Canada, and agree that it contains all necessary details for carrying out the study as described. I will conduct this protocol as outlined therein, and according to Good Clinical Practice and all applicable local regulations.

Bill Cameron____________________________
Site Qualified Investigator Name (Please Print)

____
Site Qualified Investigator Signature

____
Date

1 PROTOCOL SYNOPSIS 1
2 STUDY RATIONALE: 3
3 HYPOTHESIS**:** 3
4 OBJECTIVE(S) AND ENDPOINT(S): 3
4.1 Objectives 3
4.2 Primary endpoints: 3
4.3 Secondary endpoint: 3
5 STUDY DESIGN AND STUDY PARTICIPANTS 4
5.1 Design 4
5.2 Selection of participants 4
5.2.1 Inclusion criteria: 4
5.2.2 Exclusion criteria 5
6 STUDY TREATMENTS 5
6.1 Treatments to be administered 5
6.2 Dosage and Treatment Schedules 5
6.3 Concomitant medications 5
6.3.1 Permitted concomitant medications: 5
6.3.2 Prohibited medications: 5
6.4 Medication Adherence 6
7 STUDY PROCEDURES 6
7.1 Study Visits 6
7.1.1 Screening Visit (Between Day -30 and Day -7) 6
7.1.2 Study Day 1 6
7.1.3 Study Day 2 6
7.1.4 Study Day 3 6
7.1.5 Study Day 4 6
7.1.6 Study Day 5 7
7.1.7 Study Day 6 and 7 7
7.1.8 Study Day 8 7
7.1.9 Study Day 9 7
7.1.10 Study Day 10 7
7.1.11 Study Days 11 7
7.1.12 Study Day 12 8
7.1.13 Study Days 13 and 14 8
7.1.14 Study Day 15 8
7.1.15 Study Day 16 8
7.1.16 Study Day 30 ±3 8
7.1.17 Premature discontinuation visit 8
7.2 Observations 8
7.2.1 Drug Concentration Measurements / Pharmacokinetics 8
7.2.2 Bioanalysis 9
7.2.3 Pharmacokinetic analysis 9
7.3 Withdrawal / Discontinuation Criteria 9
8 STATISTICAL ANALYSIS 10
8.1 Statistical Analysis for Pharmacokinetics 10
9 SAFETY 10
9.1 Definitions 10
9.1.1 Adverse Events 10
9.1.2 Serious Adverse Events 10
9.1.3 Pre-existing Conditions 11
9.1.4 Procedures 11
9.1.5 Laboratory test abnormalities 11
9.2 Grading of Adverse Events 11

9.3 Causality ... 11
9.4 Expectedness ... 12
9.5 Recording, Reporting And Follow-Up Of Adverse Events ... 12
9.5.1 Non Serious Adverse Events Recording ... 12
9.5.2 Serious Adverse Events Recording ... 12
9.5.3 Adverse Events Reporting ... 12
9.5.4 Serious Adverse Events reporting ... 12
9.5.5 Adverse Events follow-up ... 13
9.5.6 Pregnancy ... 13
10 STUDY ADMINISTRATION ... 13
10.1 Applicable Standards And Regulations ... 13
10.2 Ethical Conduct Of The Study ... 14
10.2.1 Ethics Committee ... 14
10.2.2 Participant Information and Consent ... 14
10.3 Participants Compensations ... 14
10.4 Data Quality ... 14
10.4.1 Potential Risks ... 14
10.4.2 Data Safety Monitoring Board ... 14
10.4.3 Monitoring: ... 14
10.4.4 Audits ... 15
11 LENGTH OF FOLLOW-UP: ... 15
12 TIMELINES: ... 15
13 RECORD RETENTION: ... 15
14 PUBLICATION PLAN: ... 15
15 APPENDIX 1 CIOMS-I FORM ... 16
16 APPENDIX 3 SAFETY HEMATOLOGY AND BIOCHEMISTRY TESTS ... 17
17 REFERENCES ... 18

1 PROTOCOL SYNOPSIS

Long Title:	A drug interaction study investigating the effect of rifabutin on the pharmacokinetics of maraviroc
Clinical Trial Phase:	Phase I
Sponsor:	Ottawa Hospital Research Institute (OHRI)
Principal Investigator:	Bill Cameron, MD, FRCPC, Ottawa Hospital Research Institute<br>Charles la Porte, PharmD, PhD,
Background & Rationale:	The antiretroviral chemokine co-receptor 5 (CCR5) antagonist maraviroc selectively and reversibly blocks the binding of gp120 to the CCR5 receptor. This prevents the conformational changes needed for CCR5 tropic HIV-1 to enter the CD4 cells. Maraviroc is a small molecule with an absolute bioavailability of around 33%, its metabolism is mediated through CYP3A4 with a half life of around 14-18 hours, and furthermore maraviroc is a substrate of p-glycoprotein. Maraviroc is not an inducer or inhibitor of CYP3A4. In a pharmacokinetic study it was found that food decreased the AUC, but not the Cmin. No changes were noted in the viral load reduction in the fed and unfed state and therefore maraviroc can be administered with or without food. The approved standard dose of maraviroc is 300 mg BID in the absence of inhibitors or inducers of CYP3A4. In the product monograph tables are available for combinations of maraviroc and inhibitors/ inducers of CYP3A4 where the maraviroc dose needs to be adjusted to 150 mg BID or increased to 600 mg BID[1].<br>Previously a drug-drug interaction study between maraviroc and the potent CYP3A inducer rifampin resulted in around 70% decrease of maraviroc exposure and this effect could be overcome by doubling the maraviroc dose[2]. In this perspective rifabutin has not been studied as an interacting agent with maraviroc. Rifabutin is an ansamycin antibiotic used for the treatment of Mycobacterium tuberculosis and Mycobacterium avium-intracellulare complex (MAC) infections. Rifabutin is both an inducer and substrate for CYP3A4[3] Although rifabutin is a less potent inducer of CYP3A than rifampin, it is expected that maraviroc exposure will decrease as a result of the interaction with rifabutin and it might be necessary to increase the maraviroc dose to 600 mg BID when combined with rifabutin. As opposed to ritonavir boosted antiretroviral regimens maraviroc is not expected to inhibit the metabolism of rifabutin, because of the absence of CYP3A4 inhibition by maraviroc. The inhibition of rifabutin metabolism when combined with ritonavir boosted protease inhibitors frequently results in increased rifabutin toxicity and the need to decrease the dosage of rifabutin. Rifabutin has at least 5 metabolites and 25-O-desacetyl-rifabutin is a metabolite that has antibiotic activity like the parent compound and contributes up to 10% of the total activity. For this reason in assessing the effect of the drug interaction on rifabutin the pharmacokinetics of both rifabutin and 25-O-desacetyl-rifabutin will be assessed.
Investigational products:	Investigational Products will be 2 products commercially available in Canada :<br>maraviroc<br>rifabutin
Study Design:	This pharmacokinetic study will be conducted in 14 adult male and female healthy volunteers.
Intervention Description:	Treatments to be Administered<br>Substance: Maraviroc (Celsentri, MVC) tablets, 300 mg<br>Source: Commercial<br>Daily dose: 300 mg (1 tablet) twice daily at 8:00 am and 8:00 pm<br>Substance: Rifabutin (Mycobutin, RFB) capsules, 150 mg<br>Source: Commercial<br>Daily dose: 300 mg (2 capsules) once daily at 8:00 am

Sample Size:	14 participants
Study Population:	Healthy males and females, age 18 to 65,<br>seronegative for HIV, willing to stop using natural health products, certain type of food and alcohol during the study, having signed consent. Fertile female participants must be willing and able to use acceptable methods of birth control while being in the study
Study Duration:	Participants will be recruited in 8 months time. Including analysis and data report the project will take 12 months to complete
Primary Objectives:	1 The objective is to characterize the pharmacokinetic properties of maraviroc alone and when administered with rifabutin.<br>2 To assess rifabutin and 25-O-desacetyl-rifabutin pharmacokinetics compared to the literature.
Primary Outcome:	1 Maraviroc pharmacokinetics (AUC, Cmax and C12) without and with rifabutin.<br>2 Rifabutin and 25-O-desacetyl-rifabutin AUC, Cmax and C24
Secondary Outcomes:	1 safety/tolerability of the treatments

2 STUDY RATIONALE:

The antiretroviral chemokine co-receptor 5 (CCR5) antagonist maraviroc selectively and reversibly blocks the binding of gp120 to the CCR5 receptor. This prevents the conformational changes needed for CCR5 tropic HIV-1 to enter the CD4 cells. Maraviroc is a small molecule with an absolute bioavailability of around 33%, its metabolism is mediated through CYP3A4 with a half life of around 14-18 hours, and furthermore maraviroc is a substrate of p-glycoprotein. Maraviroc is not an inducer or inhibitor of CYP3A4. In a pharmacokinetic study it was found that food decreased the AUC, but not the Cmin. No changes were noted in the viral load reduction in the fed and unfed state and therefore maraviroc can be administered with or without food. The approved standard dose of maraviroc is 300 mg BID in the absence of inhibitors or inducers of CYP3A4. In the product monograph tables are available for combinations of maraviroc and inhibitors/ inducers of CYP3A4 where the maraviroc dose needs to be adjusted to 150 mg BID or increased to 600 mg BID[1].

Previously a drug-drug interaction study between maraviroc and the potent CYP3A inducer rifampin resulted in around 70% decrease of maraviroc exposure and this effect could be overcome by doubling the maraviroc dose[2]. In this perspective rifabutin has not been studied as an interacting agent with maraviroc. Rifabutin is an ansamycin antibiotic used for the treatment of Mycobacterium tuberculosis and Mycobacterium avium-intracellulare complex (MAC) infections. Rifabutin is both an inducer and substrate for CYP3A4[3] Although rifabutin is a less potent inducer of CYP3A than rifampin, it is expected that maraviroc exposure will decrease as a result of the interaction with rifabutin and it might be necessary to increase the maraviroc dose to 600 mg BID when combined with rifabutin. As opposed to ritonavir boosted antiretroviral regimens maraviroc is not expected to inhibit the metabolism of rifabutin, because of the absence of CYP3A4 inhibition by maraviroc. The inhibition of rifabutin metabolism when combined with ritonavir boosted protease inhibitors frequently results in increased rifabutin toxicity and the need to decrease the dosage of rifabutin. Rifabutin has at least 5 metabolites and 25-O-desacetyl-rifabutin is a metabolite that has antibiotic activity like the parent compound and contributes up to 10% of the total activity. For this reason in assessing the effect of the drug interaction on rifabutin the pharmacokinetics of both rifabutin and 25-O-desacetyl-rifabutin will be assessed.

We propose to study the potential drug – drug interaction of maraviroc and rifabutin in healthy adult volunteers.

3 HYPOTHESIS:

1. Rifabutin will decrease the AUC, Cmax and C12 of maraviroc.
2. Rifabutin and 25-O-desacetyl-rifabutin AUC, Cmax and C24 will be unaffected as compared to literature data (Krishna et al CMRO 2007)

4 OBJECTIVE(S) AND ENDPOINT(S):

4.1 OBJECTIVES

1. The objective is to characterize the pharmacokinetic properties of maraviroc alone and when administered with rifabutin and to assess rifabutin and 25-O-desacetyl-rifabutin pharmacokinetics compared to the literature.

4.2 PRIMARY ENDPOINTS:

1. Maraviroc pharmacokinetics (AUC, Cmax and C12) without and with rifabutin.
2. Rifabutin and 25-O-desacetyl-rifabutin AUC, Cmax and C24

4.3 SECONDARY ENDPOINT:

1. safety/tolerability of the treatments.

5 STUDY DESIGN AND STUDY PARTICIPANTS

5.1 Design

This pharmacokinetic study will be conducted in 14 adult male and female healthy volunteers. After screening, participants will be treated with maraviroc 300 mg BID for 15 days. On study day 5 after the morning dose of maraviroc, 12 hour intensive PK sampling will be performed. Samples will be taken just before and at 0.5, 1, 1.5, 2, 3, 4, 5, 6, 8, 10 and 12 hours after intake of medications. On day 6 of the study rifabutin 300 mg QD will be added to the participants' regimen for 10 days. On day 15 of maraviroc plus rifabutin treatment 12 hour intensive PK sampling will be repeated. Participants will return on day 16 for a 24 hour post dose sample for rifabutin pharmacokinetics. Participants will return to the clinic on day 30 for a final safety follow up visit. See Schedule 1 and paragraph Study Procedures for details on study design.
Meals will be standardized on PK study days. All safety and adverse event data will be collected.

Schedule 1: Study design

Day -30-7	1	2	3	4	5	6	7	8	9	10	11	12	13	14	15	16	30±3
S	V		V		PK			V		V		V			PK	V	V
	M	M	M	M	M	M	M	M	M	M	M	M	M	M	M		
						R	R	R	R	R	R	R	R	R	R		

S = screening
V = study visit
PK = intensive pharmacokinetic sampling
M = maraviroc treatment (300 mg BID)
R = rifabutin treatment (300 mg QD)

5.2 Selection of participants

14 healthy volunteers will be enrolled in this study according to the following criteria:

5.2.1 Inclusion criteria:

1. Able and willing to sign informed consent prior to any study-related activities.
2. Male or female participants between 18 and 65 years of age inclusive.
3. Body Mass Index (BMI) of 17.5 to 30.5 kg/m2; and a total body weight >50 kg (110 lbs).
4. Healthy, i.e. not suffering from an acute or chronic illness and not using medications.
5. Acceptable medical history, physical examination, and 12-lead ECG at screening.
6. Acceptable laboratory values that indicate adequate baseline organ function at screening visit.
7. Willing to stop using any herbal or natural health products for 2 weeks prior to and during the study including: Grapefruit, grapefruit juice, St. John's Wort.
8. Willingness to abstain from alcohol use for 3 days prior to and during the study.
9. Participant must practice a reliable method of birth control while they are participating in the study; for instance an intrauterine device (IUD), condom with spermicidal gel or foam, diaphragm with spermicidal gel or foam, vasectomy, tubal ligation, hysterectomy or abstinence or female must be post menopausal for at least one year.

5.2.2 Exclusion criteria

1. Have serological evidence of exposure to HIV.
2. Female patients of childbearing potential who has a positive urine pregnancy test at screening.
3. Participants not willing to use a reliable method of barrier contraception during the study.
4. Is breastfeeding.
5. Inability to adhere to protocol.
6. Use of any medications (2 weeks prior to or during the study) other than occasional use of acetaminophen.
7. Participants taking oral contraceptive medications.
8. Any condition possibly affecting drug absorption (eg, gastrectomy).
9. Patients may be excluded from the study for other reasons, at the investigator's discretion.

6 STUDY TREATMENTS

6.1 TREATMENTS TO BE ADMINISTERED

Substance:	Maraviroc (Celsentri, MVC) tablets, 300 mg
Source:	Commercial
Daily dose:	300 mg (1 tablet) twice daily at 8:00 am and 8:00 pm
Substance:	Rifabutin (Mycobutin, RFB) capsules, 150 mg
Source:	Commercial
Daily dose:	300 mg (2 capsules) once daily at 8:00 am

Participants will be provided with medications by the CIU. ViiV Healthcare will supply CIU with maraviroc. Rifabutin will be obtained commercially.

6.2 DOSAGE AND TREATMENT SCHEDULES

Maraviroc (300 mg BID) must be taken orally with approximately 250 mL of water at around 8:00 am (with breakfast) and 8:00 pm. Rifabutin (300 mg QD) must be taken orally at the same time in the morning as maraviroc.

6.3 CONCOMITANT MEDICATIONS

Participants will be asked about any medication (whether prescription or 'over the counter') or natural products that they have been taking prior to the study, or may have to take during the study. All concomitant medication use will be recorded in the CRF.

6.3.1 Permitted concomitant medications:

During the study occasional use of acetaminophen up to 1000mg per day may be used without prior consultation with the investigator.

6.3.2 Prohibited medications:

Any herbal or natural health products for 2 weeks prior to and during the study including: grapefruit, grapefruit juice, Pomelo, St. John's Wort.
Oral contraceptives are not allowed as their metabolism will be increased due to rifabutin and therefore they may not be adequate as contraception. All other co-medication should be discussed with the investigator and may lead to discontinuation of the participant.

6.4 Medication Adherence

All participants must record the date and time of administration of each dose, in the diary provided. If a dose is missed, the participant should take it as soon as it is remembered and record the actual time. In addition to that, patients will call the CIU for further instructions if required. The next dose should be taken at its original scheduled time. Non-adherent patients may be dismissed from the study at the discretion of the primary investigator. At each visit pill counts will be performed by the clinical staff. Administration of morning dose will be observed by clinical staff at Day 1, 3, 8, 10 and 12. Administration of morning and evening dose will be observed by clinical staff at Day 5 and 15.

7 STUDY PROCEDURES

7.1 Study Visits

7.1.1 Screening Visit (Between Day -30 and Day -7)

In the clinic: Fasted blood sample for hematology, chemistry and HIV test, urine sample for urinalysis, drug screening and for pregnancy test (if applicable); ECG, directed physical exam, including height and weight, and vital signs.

Participants will be contacted to confirm study dates, location and time before start of study.

7.1.2 Study Day 1

Participants will come to the clinic at 7:30.
Fasted blood sample for hematology and chemistry, urine sample for urinalysis and for pregnancy test (if applicable); ECG, weight and vital signs.
Participants will be given a standard breakfast.
Participants will be dosed with maraviroc 300 mg (8 am) (observed dose).
Participants will be given medications and instructions on how to take them at home.
Participants will be given a diary to record intake of medication at home.
At home participants will take maraviroc 300 mg around 8 pm with a snack in the evening. Participants will record times of drug intake in the diary.

7.1.3 Study Day 2

At home participants will take maraviroc 300 mg 12 hours apart at around 8am with breakfast in the morning and around 8 pm with a snack in the evening. Participants will record times of drug intake in the diary.

7.1.4 Study Day 3

Participants will come to the clinic at 7:30 and bring medications.
Fasted blood sample for hematology and chemistry, urine sample for urinalysis; vital signs.
Participants will be given a standard breakfast.
Participants will be dosed with study medication (8 am) (observed dose).
At home participants will take maraviroc 300 mg around 8 pm with a snack in the evening. participants will record times of drug intake in the diary.

7.1.5 Study Day 4

At home participants will take maraviroc 300 mg 12 hours apart at around 8am with breakfast in the morning and around 8 pm with a snack in the evening. Participants will record times of drug intake in the diary.

7.1.6 Study Day 5

Participants will come to the clinic at 7:30 and bring medications.
Fasted blood sample for hematology and chemistry, urine sample for urinalysis; ECG, directed physical exam, weight and vital signs.
Predose PK sample will be drawn.
Participants will be given a standard breakfast.
Participants will be dosed with maraviroc 300 mg (8 am) with 250mL water (observed dose).
After intake of medication participants will have to remain in an upright position for at least 2 hours. Participants will be allowed to drink after this 2 hour period and standard lunch will be served 4 hours after the morning dose and dinner will be served 10 hours post dose. Small snacks in between are allowed.
Additional PK samples will be drawn at 0.5, 1, 1.5, 2, 3, 4, 5, 6, 8, 10 and 12 hours after medication intake.
At 8 pm after the last PK sample is drawn participants will be dosed with 300 mg maraviroc after supper (observed dose).
Rifabutin medication and instructions to start taking it on day 6 will be given to participants
Participants go home.

7.1.7 Study Day 6 and 7

At home participants will take maraviroc 300 mg plus rifabutin 300 mg at around 8am with breakfast in the morning and maraviroc 300 mg around 8 pm with a snack in the evening. Participants will record times of drug intake in the diary.

7.1.8 Study Day 8

Participants will come to the clinic at 7:30 and bring medications.
Fasted blood sample for hematology and chemistry, urine sample for urinalysis; weight and vital signs.
Participants will be given a standard breakfast.
Participants will be dosed with maraviroc 300 mg and rifabutin 300 mg (8 am) (observed dose).
At home participants will take maraviroc 300 mg around 8 pm with a snack in the evening. Participants will record times of drug intake in the diary.

7.1.9 Study Day 9

At home participants will take maraviroc 300 mg plus rifabutin 300 mg at around 8am with breakfast in the morning and maraviroc 300 mg around 8 pm with a snack in the evening. Participants will record times of drug intake in the diary.

7.1.10 Study Day 10

Participants will come to the clinic at 7:30 and bring medications.
Fasted blood sample for hematology and chemistry, urine sample for urinalysis; weight and vital signs.
Participants will be given a standard breakfast.
Participants will be dosed with maraviroc 300 mg and rifabutin 300 mg (8 am) (observed dose).
At home participants will take maraviroc 300 mg around 8 pm with a snack in the evening. Participants will record times of drug intake in the diary.

7.1.11 Study Days 11

At home participants will take maraviroc 300 mg plus rifabutin 300 mg at around 8am with breakfast in the morning and maraviroc 300 mg around 8 pm with a snack in the evening. Participants will record times of drug intake in the diary.

7.1.12 Study Day 12

Participants will come to the clinic at 7:30 and bring medications.
Fasted blood sample for hematology and chemistry, urine sample for urinalysis; weight and vital signs.
Participants will be given a standard breakfast.
Participants will be dosed with maraviroc 300 mg and rifabutin 300 mg (8 am) (observed dose).
At home participants will take maraviroc 300 mg around 8 pm with a snack in the evening. Participants will record times of drug intake in the diary.

7.1.13 Study Days 13 and 14

At home participants will take maraviroc 300 mg plus rifabutin 300 mg at around 8am with breakfast in the morning and maraviroc 300 mg around 8 pm with a snack in the evening. Participants will record times of drug intake in the diary.

7.1.14 Study Day 15

Participants will come to the clinic at 7:30 and bring medications.
Fasted blood sample for hematology and chemistry, urine sample for urinalysis; ECG, directed physical exam, weight and vital signs.
Predose PK sample will be drawn.
Participants will be given a standard breakfast.
Participants will be dosed with maraviroc 300 mg and rifabutin 300 mg (8 am) with 250mL water (observed dose).
After intake of medication participants will have to remain in an upright position for at least 2 hours. Participants will be allowed to drink after this 2 hour period and standard lunch will be served 4 hours after the morning dose and dinner will be served 10 hours post dose. Small snacks in between are allowed.
Additional PK samples will be drawn at 0.5, 1, 1.5, 2, 3, 4, 5, 6, 8, 10 and 12 hours after medication intake. At 8 pm after the last PK sample is drawn participants will be dosed with 300 mg maraviroc after supper (observed dose).
Participants go home.

7.1.15 Study Day 16

Participants will come to the clinic at 7:30.
Fasted blood sample for hematology and chemistry, urine sample for urinalysis; weight and vital signs.
24 hour PK sample will be drawn.

7.1.16 Study Day 30 ±3

Participants will come to the clinic.
Fasted blood sample for hematology and chemistry, urine sample for urinalysis; ECG, directed physical exam, weight and vital signs.
End of study.

7.1.17 Premature discontinuation visit

In case of premature discontinuation, participant will be asked to come for a final visit with the Qualified Investigator to assess potential adverse events and return unused study products.

7.2 Observations

7.2.1 Drug Concentration Measurements / Pharmacokinetics

For the pharmacokinetic sampling of all analytes, 5.0 mL blood samples will be collected to obtain at least 2 mL of plasma in labeled test-tubes (K2-EDTA). The exact times of sampling will be recorded in the case report forms.

The blood samples will be mixed and stored in the refrigerator. Blood samples will be centrifuged at about 2500 g (3800 rpm for a normal laboratory centrifuge) for 10 minutes at 4°C. Plasma will be transferred to a labeled polypropylene tube and stored at ≤ -18°C within 2 hours after collection.

Blood sampling will be done according to the standard operating procedure (SOP) of the Clinical Investigation Unit. All samples will be labeled with the following information: study ID; patient study number; study day; date and time after dosing.
The exact times of sampling will be recorded in the case report forms.

7.2.2 Bioanalysis

All applicable plasma samples will be analyzed for maraviroc and rifabutin and 25-O-desacetyl-rifabutin. All available plasma samples from this study will be analyzed using a validated High Performance Liquid Chromatography (HPLC) tandem Mass Spectrometry (MS/MS) method. Sample pre-treatment will consist of protein precipitation with methanol, after which the supernatant will be used for injection. A multipoint ($n \geq 6$) calibration curve will be generated for each analytical run and will be used to calculate the concentration of the compounds of interest in the samples. A set of Quality Control samples (low, medium, high) will be analyzed at the start and at completion of each analytical run. Methods will be based on a previously published method for maraviroc[4].

7.2.3 Pharmacokinetic analysis

Pharmacokinetic parameters for maraviroc and rifabutin and 25-O-desacetyl-rifabutin will be calculated using WinNonlin version 5.0.1 (Pharsight Corporation 2005) or comparable techniques. Based on the individual plasma concentration-time data, the following pharmacokinetic parameters will be determined in a non-compartmental analysis: AUC, (mg*h/L) the area under the plasma concentration-time curve; Cmax, (mg/L) the maximum plasma drug concentration; C12, (mg/L), the 12 hour post dose plasma concentration for maraviroc and C24, (mg/L), the 24 hour post dose plasma concentration for rifabutin Tmax, (h) the time to reach maximum plasma drug concentration; Cl/F, (L/h) apparent clearance; and Vd/F, (L) volume of distribution.

7.3 Withdrawal / Discontinuation Criteria

The trial as a whole will be terminated prematurely if more than 33% of the subjects show drug-related and clinically relevant adverse events of severe intensity.

Participants have the right to withdraw from the study treatment at any time for any reason. Investigator has the right and obligation to withdraw Participants from the study treatment in the event of:

1 Adverse event which would, in the judgment of the investigator require discontinuation of study product(s)
2 Request by the participant or of their legally authorized representative (consent withdrawal)
3 Non compliance to the study protocol
4 Participants needs additional medication that would interfere with the study
5 Pregnancy
6 Participant is lost to follow-up
7 The sponsor, or the principal investigator or the Data and Safety Monitoring Board or a government agency such as Health Canada cancels the study

When a participant withdraws consent the following situations will be encountered:
1-Participant never starts protocol treatment.
2-Participant's withdrawal of consent after starting the protocol treatment: The investigator or delegate should make any effort to contact the participant to determine as completely as possible the reason for the withdrawal and to schedule an end-of-study visit for assessing potential adverse events. The participant will be asked to return unused study products.
3-Participants who have been withdrawn from the study cannot be re-included in the study. Their inclusion and treatment number must not be reused.

8 STATISTICAL ANALYSIS

Descriptive statistics will be produced for demographics, safety and pharmacokinetic parameters for all study participants. Participants may be excluded from the statistical analysis of pharmacokinetics if no reliable pharmacokinetic parameters can be determined as a result of vomiting or fever after drug administration on PK study days.

8.1 Statistical Analysis for Pharmacokinetics

In this trial, evaluation of AUC, Cmax and C12 of maraviroc is the main objective. These parameters are considered to be the primary characteristics of the extent and rate of drug absorption. Individual results will be presented for reference treatment (i.e., maraviroc alone) and test treatment (i.e.,maraviroc plus rifabutin) together with the ratios test treatment/reference treatment. The geometric mean and the coefficient of variation corresponding to mean and standard deviation in the logarithmically transformed domain will be given for test and reference treatments. The geometric mean ratio and 90% classical confidence interval will be calculated for the treatment ratio: test treatment/reference treatment.

Treatments will be considered bioequivalent if the respective 90% classical confidence intervals for AUC, Cmax and C12 are included within the bioequivalence range of 80 to 125%.

The PK parameters of rifabutin in combination with maraviroc will be compared to those of rifabutin alone as published in the literature (Krishna et al CMRO 2007)[5] using standard T-tests.

9 SAFETY

Adverse events will be documented in the CRF on all visits. Participants will be monitored for adverse effects on PK sampling days. All adverse event data and clinical laboratory data will be included in the study report.
Safety blood tests as mentioned in appendix 3 will be collected regularly during the study. During the entire trial, a total volume of approximately 240 mL blood will be sampled from each participant. This volume includes all samples for pharmacokinetics and safety assessments.
The use of rifabutin may impart a red-orange colour to the urine and possibly to skin and body secretions. Contact lenses, especially the soft variety, may be permanently stained[3]. It is therefore recommended not to use contact lenses for the duration of the treatment with rifabutin.

9.1 Definitions

9.1.1 Adverse Events

An Adverse Event (AE) is any untoward medical occurrence in a participant or clinical investigation participant administered a pharmaceutical product, which does not necessarily have to have a causal relationship with this treatment.

An adverse event can therefore be:

- any unfavorable and unintended sign (including an abnormal laboratory finding);
- symptom or disease temporally associated with the use of a medicinal product, whether or not considered related to the medicinal product;
- pre-existing symptoms or conditions which worsen during a study.

9.1.2 Serious Adverse Events

Serious Adverse Event (SAE)

1 Results in Death
2 Is life threatening

3 Requires participant hospitalization or prolongation of existing hospitalization
4 Results in persistent or significant disability/incapacity
5 Is a congenital anomaly/ birth defect

Some conditions may not be immediately life threatening or require hospitalization. Should the investigator feel that the event may jeopardize the participant or may require intervention to prevent more serious outcomes, then it should be treated as serious. Hospitalizations for routine procedures and investigations are not considered a SAE in this protocol.

9.1.3 Pre-existing Conditions

In this trial, a pre-existing condition (i.e., a disorder present before the adverse event reporting period started) should not be reported as an adverse event unless the condition worsens during the adverse event reporting period.

9.1.4 Procedures

Diagnostic and therapeutic non-invasive and invasive procedures, such as surgery, should not be reported as adverse events. However, the medical condition for which the procedure was performed should be reported if it meets the definition of an adverse event. For example, an acute appendicitis that begins during the adverse event reporting period should be reported as the adverse event and the resulting appendectomy noted under Comments.

9.1.5 Laboratory test abnormalities

Laboratory test value abnormalities will be reported on the AE electronic database as adverse events, if they satisfy one or more of the following conditions for clinical significance:

1. accompanied by clinical symptoms
2. leading to a change in study medication (e.g. dose modification, interruption or permanent discontinuation)
3. requiring a change in concomitant therapy (e.g. addition of, interruption of, discontinuation of, or any other change in a concomitant medication, therapy or treatment).

Please note: any laboratory result abnormality fulfilling the criteria for a serious adverse event (SAE) should be reported as such, in addition to being recorded as an adverse event in the CRF.

9.2 Grading of Adverse Events

All adverse events will be graded by the Investigator using the five-point scale below.

1=Mild	discomfort noticed but no disruption of normal daily activity
2=Moderate	discomfort sufficient to reduce or affect daily activity
3=Severe	inability to work or perform normal daily activity
4=Life Threatening or Disabling	represents an immediate threat to life
5=Death	related to AE

9.3 Causality

Relationship of all adverse events and serious adverse events to the study interventions (causality) should be assessed by the investigator as follows:

- **Unrelated:** There is not a reasonable possibility that the adverse event may have been caused by the study drug.
- **Possibly related**: The adverse event may have been caused by the study drug, however there is insufficient information to determine the likelihood of this possibility.
- **Related**: There is a reasonable possibility that the adverse event may have been caused by the study drug.

9.4 EXPECTEDNESS

Adverse events and serious adverse events will also be assessed according to the following categories:

- **Expected (anticipated):** the event is identified in nature, severity, or frequency in the product monograph.
- **Unexpected (unanticipated):** the event is not identified in nature, severity, or frequency in the product monograph.
- **More prevalent:** the event occurs more frequently than anticipated or at a higher prevalence than expected.

9.5 RECORDING, REPORTING AND FOLLOW-UP OF ADVERSE EVENTS

9.5.1 Non Serious Adverse Events Recording

All new adverse events experienced after the participant received the study treatment should be recorded in the CRF. AEs (any untoward medical occurrence in trial Participant) do not necessarily have to have a causal relationship with treatment.

The investigator and delegates will record all directly observed adverse events and all adverse events spontaneously reported by the participant. In addition, each participant will be questioned about adverse events at each visit.

9.5.2 Serious Adverse Events Recording

All serious adverse events or abnormal laboratory test value, expected or unexpected, occurring after the participant signed the informed consent form, will be recorded in the Case Report Form.

9.5.3 Adverse Events Reporting

The PI will be responsible for reporting the AES to the OHREB, Health Canada, Pfizer and the Data and Safety Monitoring Board (DSMB) as follows:

Grade 3, or greater, adverse events occurring in a participant that received at least one dose of one of the study drugs will be reported immediately to the Chair of the DSMB)

Expected Adverse Events should be reported to the OHREB in summary format on an annual basis in the annual renewal report.

- **Unexpected Adverse Events** should be reported within 15 days of the event or notification of the event.

- **Adverse events** will be summarized in a table, indicating the severity causality and expectedness, and provided to the Data and Safety Monitoring Board a minimum of two weeks before each DSMB meeting.

All adverse events will be summarized in a table, and provided to the DSMB before DSMB meetings.

9.5.4 Serious Adverse Events reporting

SAEs require prompt or immediate reporting to PI or designee, The PI will be responsible for reporting the SAEs to Health Canada, the OHREB, the Investigator's Review Committee and the DSMB.

Reporting to the DSMB
All SAEs, whether related to the study interventions or not, will be reported immediately to Chair of the DSMB.
All SAEs, whether related to the study interventions or not will be summarized in a table, and provided to the DSMB before the scheduled DSMB meeting.
Reporting to the OHREB
All SAEs, whether related to the study interventions or not, will be reported to the OHREB as follows.

Serious and Expected Events: If there is evidence of an increased rate of serious but expected AEs, a report should be submitted to the OHREB in a timely fashion,.

Serious and Unexpected Events: reported within 7 days of the event or notification of the event. If the event is fatal or life-threatening, reporting should be immediate when possible and, in any event, within 7 days after notification of the event.

Reporting to Health Canada

Serious adverse events that are both unexpected and related or possibly related are participant to expedited reporting to Health Canada. SAE that are expected or that are unrelated to the study drug are not reportable.
Report must be filed in the cases:

where the SAE is neither fatal nor life-threatening, within 15 days after becoming aware of the information

where it is fatal or life-threatening, immediately where possible and, in any event, within 7 days after becoming aware of the information

within 8 days after having informed Health Canada of the SAE, the principal investigator will submit as complete as possible, a report which includes an assessment of the importance and implication of any findings

Each SAE which is participant to expedited reporting should be reported individually using the CIOMS-I form (see Appendix 2)

Any updated follow up information that becomes available regarding the SAE should be reported in a follow up report

Reporting to Pharmacovigilance Group at Pfizer

The principal Investigator will provide the Pharmacovigilance Group at Pfizer with copies of all serious adverse experiences, which are possibly related, or related to use of boceprevir within two working days, and a copy of the reports provided to Health Canada, within 24 h.

9.5.5 Adverse Events follow-up

Follow-up of adverse events considered related or possibly related to study drugs should continue until they have returned to baseline status or stabilized or the causal relationship has been changed from related to unrelated to study drug.

9.5.6 Pregnancy

Female participants are required to use adequate contraception methods as part of the entry criteria.
If any trial participant becomes or is found to be pregnant while receiving a study drug or within 30 days of discontinuing the study drugs, the pregnancy will be reported in the same way as serious adverse events i.e. immediately and using the SAE Report Form
Pregnancies are followed by the investigator until completion of the pregnancy to learn the outcome as congenital anomaly/birth defect is a category of SAE.
Pregnancy will be reported to Pfizer as per procedure outlined in the grant agreement.

10 STUDY ADMINISTRATION

10.1 Applicable Standards And Regulations

The trial will be performed in compliance with the Canadian Tri-Council Policy Statement version 2 (TCPS2), the World Medical Association Declaration of Helsinki, adopted by the 18th World Medical Assembly, Helsinki, Finland, June 1964, and last amended by the 52nd WMA General Assembly, Edinburgh, Scotland, October 2000, the Canadian Food and Drug Regulations, Division 5 Part C, the Canadian Ontario's Personal Health Information. Protection Act (PHIPA), and the ICH Good Clinical Practices (GCP).

10.2 Ethical Conduct Of The Study

10.2.1 Ethics Committee

The trial may only be initiated after the Investigator has obtained written approval of the protocol, Informed Consent form and other study documents, and any amendments (if applicable), by the Ottawa Hospital Research Ethics Board (OHREB). Changes in protocol (amendments) must be submitted to the OHREB for approval. Reports on, and reviews of, the trial, its safety aspects and its progress will be submitted to the OHREB by the Investigator at intervals according to their guidelines.

10.2.2 Participant Information and Consent

The Investigator (or another authorized person from the clinical staff) will explain to each potential Participant the aims, methods, reasonably anticipated benefits and potential hazards of the trial and any discomfort in written form and by verbal explanation in non-technical terms.
After this explanation and prior to performance of any trial related activity, Participants will give their informed consent by completing the consent form in the written participant information. Both the Participant and the Investigator sign and date the form. The Participant will receive a copy of the completed informed consent form.

10.3 Participants Compensations

Study participants will be compensated up to a maximum of $1020 for this study. This compensation may be prorated if they decide to discontinue early. The compensation is for their time investment as well as expenses for the study.

10.4 Data Quality

10.4.1 Patient Safety Monitoring

Patients' safety monitoring during the conduct of the trial, and the management of possible associated expected and/ or unexpected adverse drug reaction, is to be carried out by Drs Bill Cameron and / or Jonathan Angel, both Ontario licensed physicians.

10.4.2 Data Safety Monitoring Board

The Ottawa Method Centre will appoint and oversee the DSMB for the RIFAMARA study, under the direction of Dr. Tim Ramsay (Scientific Director). The DSMB will meet once soon after initiating the conduct of the PK trial, and as need be thereafter.
Additional meetings may be called by Dr. Tim Ramsay if a safety concern should arise. Meetings may be face to face or virtual, but all discussions and decisions will be documented in writing. The Investigator will be responsible for immediately informing the DSMB Chair of becoming aware of any grade 3 and greater toxicity and serious adverse event occurring in a participant who has received at least one dose of any of the study treatments.

All reports provided to the Principal Investigator by the DSMB will be immediately forwarded to the Chair of the OHRI Clinical Research Monitoring Committee.

10.4.3 Monitoring:

The investigator (or his/her designee) agrees to cooperate with the OHRI monitor to schedule at least one monitoring visit during the course of the study and to ensure that any problems detected in the course of this monitoring visit are resolved. This monitoring by OHRI does not replace the routine quality control to be performed by the Principal Investigator or his designee.

Routine quality control will be completed by the Principal Investigator and his designee at Clinical Investigation Unit to ensure that the study is being run according to the protocol. The routine monitoring of the study will include:

- Verification of all inclusion and exclusion criteria to confirm that only eligible participants are participating in the trial
- Verification of source data to ensure accuracy of study data
- Verification that all adverse events are recorded, assessed and reported according to the protocol
- Verification of the case report forms to ensure that they are being completed according to the protocol

10.4.4 Audits

The Investigator should understand that OHRI or its designees, after appropriate notification may audit the study for Quality Assurance purposes. The verification of the electronic database must be by direct inspection of source document.

11 LENGTH OF FOLLOW-UP:

Approximately 4 weeks per treatment cohort.
Participants experiencing an adverse event related or possibly related to the study products will be followed by the qualified investigator until resolution of the adverse event.

12 TIMELINES:

14 Participants will be recruited in 10 months time. Including analysis and data report the project will take 16 months to complete.

13 RECORD RETENTION:

To comply with Health Canada regulations, the trial related records will be retained for 25 years. Records will be kept to enable linkage of participants' identity to CRF data (master log). This includes sufficient information from hospital and clinic records such as all original signed informed consent forms, source records, and detailed records of cell manufacturing. After 25 years, all study records will be destroyed by shredding or incineration

14 PUBLICATION PLAN:

The results of the study will be presented at an international meeting as well as in an international pharmacology or infectious disease journal

15 APPENDIX 1 CIOMS-I FORM

CIOMS FORM

SUSPECT ADVERSE REACTION REPORT

I. REACTION INFORMATION

1. PATIENT INITIALS (first, last)	1a. COUNTRY	2. DATE OF BIRTH Day / Month / Year	2a. AGE Years	3. SEX	4-6 REACTION ONSET Day / Month / Year	8-12 CHECK ALL APPROPRIATE TO ADVERSE REACTION
7 + 13 DESCRIBE REACTION(S) (including relevant tests/lab data)						☐ PATIENT DIED<br>☐ INVOLVED OR PROLONGED INPATIENT HOSPITALISATION<br>☐ INVOLVED PERSISTENCE OR SIGNIFICANT DISABILITY OR INCAPACITY<br>☐ LIFE THREATENING

II. SUSPECT DRUG(S) INFORMATION

14. SUSPECT DRUG(S) (include generic name)		20 DID REACTION ABATE AFTER STOPPING DRUG? ☐ YES ☐ NO ☐ NA
15. DAILY DOSE(S)	16. ROUTE(S) OF ADMINISTRATION	21. DID REACTION REAPPEAR AFTER REINTRODUCTION? ☐ YES ☐ NO ☐ NA
17. INDICATION(S) FOR USE		
18. THERAPY DATES (from/to)	19. THERAPY DURATION	

III. CONCOMITANT DRUG(S) AND HISTORY

22. CONCOMITANT DRUG(S) AND DATES OF ADMINISTRATION (exclude those used to treat reaction)

23. OTHER RELEVANT HISTORY (e.g. diagnostics, allergics, pregnancy with last month of period, etc.)

IV. MANUFACTURER INFORMATION

24a. NAME AND ADDRESS OF MANUFACTURER	
	24b. MFR CONTROL NO.
24c. DATE RECEIVED BY MANUFACTURER	24d. REPORT SOURCE ☐ STUDY ☐ LITERATURE ☐ HEALTH PROFESSIONAL
DATE OF THIS REPORT	25a. REPORT TYPE ☐ INITIAL ☐ FOLLOWUP

16 APPENDIX 3 SAFETY HEMATOLOGY AND BIOCHEMISTRY TESTS

Hematology:

Hemoglobin
Hematocrit
Total white cell count
Differential white cell count
Red blood cell count
Mean Corpuscular Volume
Mean Corpuscular Hemoglobin
Mean Corpuscular Indirect
Hemoglobin Concentration
Platelet count

Biochemistry:

Glucose
Sodium
Potassium
Chloride
Blood Urea Nitrogen (BUN)
Creatinine

Alanine Aminotransferase- ALT
Aspartate Aminotransferase- AST
Gamma-Glutamyl Transferase-GGT

Total Bilirubin

17 REFERENCES

1. *Maraviroc Product Monograph*, in *Pfizer*. 2009. p. 29.
2. Abel, S., et al., *Effects of CYP3A4 inducers with and without CYP3A4 inhibitors on the pharmacokinetics of maraviroc in healthy volunteers.* Br J Clin Pharmacol, 2008. **65 Suppl 1**: p. 38-46.
3. *Rifabutin Product Monograph*, in *Pharmacia & Upjohn*. 2008. p. 1-10.
4. Brewer, E., et al., *An LC-MS-MS method for quantitative determination of maraviroc (UK-427,857) in human plasma, urine and cerebrospinal fluid.* Biomed Chromatogr, 2010. **24**(12): p. 1316-23.
5. Krishna, G., et al., *Evaluation of the pharmacokinetics of posaconazole and rifabutin following co-administration to healthy men.* Curr Med Res Opin, 2007. **23**(3): p. 545-52.
